# Binary Polymeric Surfactant Mixtures for the Development of Novel Loteprednol Etabonate Nanomicellar Eyedrops

**DOI:** 10.3390/ph16060864

**Published:** 2023-06-10

**Authors:** Silvia Tampucci, Daniela Monti, Susi Burgalassi, Eleonora Terreni, Valentina Paganini, Mariacristina Di Gangi, Patrizia Chetoni

**Affiliations:** 1 Department of Pharmacy, University of Pisa, 56126 Pisa, Italy; 2 Italian Inter-University Center for the Promotion of the 3Rs in Teaching and Research, University of Pisa, 56122 Pisa, Italy

**Keywords:** loteprednol etabonate, Kolliphor^®^ HS-15, D-alpha-tocopheryl polyethylene glycol 1000 succinate, TPGS, ocular drug delivery, mixed nanomicelles, critical micellar concentration

## Abstract

The treatment of several ocular inflammatory conditions affecting different areas of the ocular globe involves the administration of topical ophthalmic formulations containing corticosteroids. This research was aimed at evaluating the solubilising efficacy of 5.0% *w*/*w* of different binary mixtures of commercial amphiphilic polymeric surfactants with the purpose of obtaining nanomicellar solutions containing a high amount of loteprednol etabonate (LE). The selected LE-TPGS/HS nanomicelles, containing 0.253 mg/mL of the drug, had a small size (=13.57 nm) and uniform distribution (Polydispersity Index = 0.271), appeared completely transparent and perfectly filterable through 0.2 μm membrane filter, and remained stable up to 30 days at 4 °C. The critical micellar concentration (CMC_TPGS/HS_) was 0.0983 mM and the negative value of the interaction parameter between the polymeric-surfactant-building unit (β_TPGS/HS_ = −0.1322) confirmed the ability of the polymeric surfactants to interact, favouring the dissolution of LE into nanomicelles. The disappearance of the endothermic peak of LE in the DSC analysis confirmed the interactions of LE with the polymeric surfactants. LE-TPGS/HS produced in vitro LE which sustained diffusion for 44 h (more than 40% of encapsulated LE). Furthermore, the lack of a significant cytotoxic effect on a sensitive corneal epithelial cell line makes it a candidate for further biological studies.

## 1. Introduction

The treatment of several ocular pathologies characterised by a marked inflammatory component, at both the anterior and posterior segment level, involves the administration of topical ophthalmic formulations containing corticosteroids [1,2]. The application of these drugs ranges from the treatment of inflammation and pain generally associated with post-operative ocular surgery (e.g., cataract surgery), to chronic pathological situations (e.g., dry eye syndrome) [3]. However, the application of corticosteroids in the ocular field is generally associated with a number of serious adverse events and risks for patients, especially when the drugs are administered over a prolonged period of time. The continuous instillation of eye drops containing corticosteroids increases the risk of bacterial, viral and fungal infections, rising intraocular pressure (IOP), posterior subcapsular cataract formation and the delayed healing of ocular tissues [4]. Therefore, the choice of appropriate corticosteroid molecules is fundamental for a specific therapy and must take into account, in addition to the pharmacological potential, several technological/formulative features that generally influence the bioavailability of drugs. Indeed, the presence of physiological ocular defence mechanisms such as blinking and increased tear flow with the consequently quick drainage of drugs from the precorneal area have a substantial influence in the case of eye drop suspensions [5,6]. As such, the limited ocular bioavailability of corticosteroids, generally formulated as suspensions, is often related to a poor drug dissolution rate in the tear fluid, a high precorneal dilution/elimination rate due to tear turnover and poor drug penetration/absorption into ocular tissues [4,7]. Although with the use of micronised suspensions, the increase in viscosity and/or mucoadhesiveness lead to a higher concentration of dissolved drug and the improved bioavailability of the corticosteroids, respectively, the influence of these formulation approaches is still poorly verifiable from a biopharmaceutical point of view [8,9,10,11]. Therefore, the proposal of a technological approach for the formulation of insoluble drugs, such as corticosteroids, in the form of an ocular suspension can represent a winning strategy. On the other hand, a technological resolution based on simple particle size reduction can often be associated with the need for frequent instillations of the formulation with poor patient compliance and the possible appearance of adverse effects due to non-productive systemic drug absorption through the nasolacrimal duct [12].

Furthermore, the use of eye suspensions implies other technological problems, such as a poor homogeneity of particle dimensions, a high sedimentation rate and/or aggregation of suspended particles, the scarce availability of suitable sterilisation methods and the excessive cost of the products due to the high amount of drug required to reach the therapeutically active dose for the suspensions. 

Finally, the failure of many therapies involving traditional hydrophobic drug suspensions is most likely due to the insufficient retention and accumulation of drug at the target site, resulting in suboptimal therapeutic levels. The significant advances in the development of nano-sized (1–200 nm) ocular drug delivery systems over the past decades may provide hints to solve these issues. The use of nanomicelles obtained by self-assembling amphiphilic molecules in concentrations above the critical micellar concentration (CMC) has attracted considerable interest. In particular, the use of different molecules, such as binary mixtures of surfactants and/or amphiphilic polymeric surfactants, favours the drug solubilisation process and stabilises delivery systems [13,14,15,16].

Loteprednol etabonate (LE) is a carbon-20 ester-based corticosteroid for ophthalmic use that, by applying the principle of retro-metabolic drug design, allows an appropriate balance between several biopharmaceutical properties such as lipophilicity, distribution in ocular tissues, binding to glucocorticoid receptors, rate of de-esterification into an inactive metabolite and good local activity without systemic side effect induction [17]. Suspensions containing 0.5% or 0.2% *w*/*w* LE were approved by the US Food and Drug Administration (FDA) in 1998 for the topical management of many ocular inflammatory diseases with the commercial names Lotemax™ and Alrex™ (Bausch & Lomb, Inc., Rochester, NY, USA), respectively. They are active in reducing a series of steroid-responsive inflammatory conditions of the eye, such as giant papillary conjunctivitis, acute anterior uveitis, inflammation following cataract extraction with intraocular lens implantation and seasonal allergic conjunctivitis. Other formulations have been developed and in some cases marketed: (i) a non-settling gel formulation with 0.55% of LE in 2012, able to drop a uniform dose without shaking the bottle before administration [18]; (ii) a 1% LE suspension (Inveltys™, Kala Pharmaceuticals, Arlington, MA, USA) in 2018; and (iii) a 0.38% submicron LE ophthalmic gel (Lotemax SM™, Bausch & Lomb Inc., Rochester, NY, USA) in 2019 [19]. Ultimately, the technological strategies address the use of micronised LE, in the attempt to maintain the stability of the suspension and to improve the ocular bioavailability and the therapeutic efficacy of the drug. Recently, Kala Pharmaceuticals launched Eysuvis™ in the U.S. (2021), consisting of a 0.25% LE ophthalmic suspension, whose approval by the FDA was based on clinical results of short-term treatment (up to 2 weeks) showing an improvement in both signs and symptoms of dry eye disease due to the novel mucus-penetrating nanoparticle technology [20,21]. Regardless of the approved products some other technological strategies have been evaluated for the formulation of LE-based eye drops such as the cationic nanoemulsified in situ ophthalmic gel proposed by Patel et al. [22], to improve the retention time of formulations and LE permeability; complexation using various cyclodextrins to increase the LE aqueous solubility and stability [23]; the use of nanoparticle technologies to improve LE corneal permeability [24]; the development of a novel mucoahesive shell-crosslinked nanogel system for therapeutic effect enhancement [25].

In the light of this scientific background, the aim of this research was to evaluate the solubilising efficacy of binary mixtures of commercial amphiphilic polymeric surfactants with the final purpose of obtaining nanomicellar solutions with a high amount of encapsulated LE in their lipophilic core. The selected amphiphilic polymeric surfactants had similar hydrophilic–lipophilic balance (HLB) values and were used at the same weighted concentration in the binary mixtures, in each case above their CMC. The nanomicellar systems were characterised for clarity, size distribution, thermal behaviour, amount of solubilised LE and physico-chemical stability. In view of their ocular application, a cytotoxicity study on rabbit epithelial cell lines and an in vitro LE dissolution test were carried out.

Finally, the results were analysed on the basis of regular solution theory, to evaluate in detail the interaction parameters between the different binary polymeric compositions.

## 2. Results and Discussion

### 2.1. Development of LE-Loaded Mixed Nanomicelles (LE-MixNano)

The choice of excipients for the development of ocular formulations requires both theoretical considerations of known data and specific evidence obtained with experimental data related to the selected drug and/or type of application. In fact, excipients are essential components of ophthalmic formulations capable of favouring both the development of different dosage forms (eye drops, suspensions, ointments) and the specific technological/biopharmaceutical drug properties such as solubility and permeability, the pharmacokinetic profile, toxicity, and the stability of the drug itself or of the pharmaceutical system.

For this study, we selected a group of adjuvants with homogeneous HLB values, often used in the solubilisation process of lipophilic drugs mainly for oral and parenteral administration, although their use may also concern the ocular application. 

**Figure 1:**
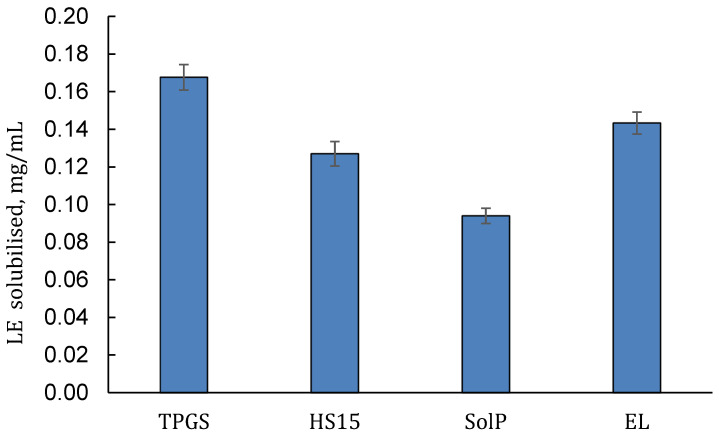
Solubility of LE in presence of 5.0% *w*/*w* of different polymeric surfactants (Isodel^®^ Vitamin E TPGS 1000 (TPGS), Kolliphor^®^ HS-15 (HS), Soluplus^®^ (SolP) and Kolliphor^®^-EL (EL)) (mean ± SD, n = 3).

The results of the first preliminary screening of the solubilisation power of several selected polymeric surfactants used in concentrations of 5% *w*/*w* are displayed in Figure 1, where the amount of solubilised LE (LE-In) in Sorensen phosphate-buffered solution (PBS, pH 7.4) is reported for each selected surfactant. In all cases, the concentrations of each polymeric surfactant were noticeably above the CMC values. Isodel^®^ Vitamin E TPGS 1000 (TPGS) seems the more promising ingredient since it determined a remarkable LE solubilisation, with a more-than-330-fold improvement in LE water solubility, from 0.0005 mg/mL [22] to 0.168 ± 0.007 mg/mL.

A substantial increase in LE-In (0.143 ± 0.006 mg/mL) was also observed by using 5% *w*/*w* Kolliphor^®^-EL (EL), while a lower LE solubility was obtained by using both Kolliphor^®^ HS-15 (HS) and Soluplus^®^ (SolP). The amount of LE was 0.127 ± 0.007 and 0.094 ± 0.004 mg/mL, with an increase of more than 254 and 188 times the LE solubility, for HS and SolP, respectively.

The results of the preliminary screening agree with those of other scientific studies. Probably, the strong amphiphilic character of the selected substances allowed the formation of nanomicelles with a hydrophobic core that was useful as reservoir for LE and consequently the development of LE eye drops. In addition, nanomicelles are more promising systems for ocular delivery as they offer many other advantages, as suggested by Sharma et al. [26]. In fact, TPGS nanomicelles, in addition to having an enhancing effect on LE solubility, possess a potential beneficial effect on various ophthalmic pathologies such as age-related macular degeneration, uveitis, glaucoma, and cataract [27,28,29,30].

In order to obtain an improvement in LE encapsulated into the hydrophobic core of the micelles, the mixed micelles strategy was evaluated. In fact, synergistic interactions between different polymeric surfactants in the binary mixed systems may result in successful applications, with positive repercussions on the stability of the nanomicelles and/or on the increase in the amount of the solubilised drug and/or optimisation of the dimensions of the mixed nanomicelles. 

In our study, nanomicelles developed by mixing the same concentration (5.0% *w*/*w*) of the selected polymeric surfactants produced small and homogeneously distributed (polydispersity index—PI < 0.343) nanomicelles with a mean size ranging from 12.03 nm to 17.93 nm, in particular in the presence of TPGS (see Table 1). On the other hand, the use of SolP allowed the production of nanomicelles of a greater size and with higher PI values (size: 85.13 nm and 68.27 nm; PI: 0.507 and 0.471 for LE-HS/SolP and LE-SolP/EL, respectively). 

The highest amount of LE encapsulated in mixed nanomicelles was produced in the presence of TPGS with more than a 500-fold improvement in LE-In for LE-TPGS/HS (0.253 ± 0.007 mg/mL LE concentration) and with the highest encapsulation efficiency (LE-EE% = 25.3). In each case, statistically significant differences in LE-In were obtained with the mixed polymeric surfactant nanomicelles based on TPGS/HS and TPGS/EL (*p* < 0.05, ANOVA followed by Tukey’s multiple comparison test). 

However, exclusively LE-TPGS/HS nanomicelles appeared completely transparent and perfectly filterable through a 0.2 μm membrane filter, while all the other formulations, included LE-TPGS/EL, appeared opaque and often had poor filterability. This behaviour reduces the peculiarities required for an ophthalmic formulation. In fact, the use of sterilisation methods other than filtration can modify the formulation stability, with drug precipitation and/or phase separation. On the other hand, even the LE-HS/EL nanomicellar formulation appeared sufficiently transparent, with high filterability, and with small dimensions (13.47 ± 0.31 nm) but LE-In was significantly lower (0.129 ± 0.002 mg/mL) and not statistically different from that of LE-EL/SolP formulation.

PI values represent a useful index of nanomicelle uniformity as they are related to the nanomicelle size distribution. In general, the nanomicellar systems containing SolP showed higher PI values, while the other binary surfactant combinations showed lower PI values and were consequently homogeneous in size. The lowest PI value was obtained for LE-HS/EL, while for the TPGS containing nanomicelles the PI values ranged between 0.271 and 0.343.

Low PI values obtained for LE-HS/EL reflect a typical monodisperse nanomicellar system with great stability, while highest PI values such as those measured for LE-HS/SolP and LE-EL/SolP indicate poor particle stability and the presence of any aggregates [31].

### 2.2. CMC Determinations and Synergistic Effect of Surfactant Mixtures

The critical micellar concentration experimental (CMC_exp_) values measured via the optical method of the pendant drop for each different single substance are reported in Table 2. The surface tension for the different polymeric surfactants showed a regular decrease in the premicellar region, which sharply reduced with the increase in the polymeric surfactant concentration. CMC_exp_ values can be estimated as the concentration corresponding to the intersection between the linear fitting of the rapidly decreasing portion of surface tension logarithm of the concentration curve and the almost-horizontal portion for the lower surface tension values.

The CMC_exp_ values for each single polymeric surfactant were almost in agreement with the theoretical (CMC_theor_) values reported in the data sheet for the pure amphiphilic substances (Table 2). In most cases, a slight shift towards higher values of CMC were observed for the CMC_exp_ values with respect to the theoretical ones, apart for the HS polymeric surfactant. Generally, the presence of electrolytes produces a more thermodynamically favourable condition for CMC reduction, probably due to the repulsion between the ionic headgroups of polymeric surfactants. This behaviour appears more pronounced at higher salt concentrations and/or in the presence of non-ionic surfactants [32]. However, in our study, the selection of PBS instead of water, as a medium for the development of ophthalmic preparations, seemed to not determine an appreciable decrease in CMC.

In addition, by applying the ideal mixing theory proposed by Clint [33], the theoretical ${\mathrm{CMC}}_{\mathrm{mix}}^{*}$ and $X_{1}^{*}$ mole fraction values were calculated for the selected binary mixtures (Table 3), applying Equations (2) and (3), reported in Section 3.6.4. 

In general, the use of different polymeric binary surfactant mixtures results in lower ${\mathrm{CMC}}_{\mathrm{mix}}^{*}$ values with respect to the first component of the binary mixture. This behaviour was more evident in presence of SolP. In fact, a significant reduction in ${\mathrm{CMC}}_{\mathrm{mix}}^{*}$ was observed in all binary mixture containing SolP; this was probably correlated to the high theoretical molar fraction of SolP in the mixed micelles that caused a greater contribution to the ${\mathrm{CMC}}_{\mathrm{mix}}^{*}$ values. 

Indeed, even within the approximation that the Clint’s theory of ideal behaviour can provide, for all the binary mixtures containing SolP, $X_{1}^{*}$ values independent of the molar fraction values in the bulk (α_1_) ranged from 2.18 × 10^−5^ to 4.51 × 10^−4^. The presence of a high fraction of the SolP allows the formation of a hydrophobic core useful for the incorporation of a high amount of the lipophilic drug as suggested by Bernabeu et al. [34]. In our study, notwithstanding the increase in the nanomicelle sizes (85.13 and 68.27 nm, respectively, for LE-HS/SolP and LE-EL/SolP) that is typical for this hydrophobic block copolymer, the capability to enhance the LE solubility remained low.

Regardless, a significant reduction in the ${\mathrm{CMC}}_{\mathrm{mix}}^{*}$ values was observed in all cases, except for the binary mixture HS/EL. This represents an advantageous result, since a low CMC value ensures stability, preventing the loss of the encapsulated drug during dilution with tear lacrimal fluid for an ophthalmic preparation.

On the basis of the collected results, the more promising binary mixture of polymeric surfactants appeared to be the one based on TPGS/HS. Nevertheless, there were statistically significant differences in the amount of LE encapsulated for the majority of polymeric mixtures (*p* < 0.05, ANOVA followed by Tukey’s multiple comparison test), except for TPGS containing nanomicelles (LE-TPGS/SolP vs. LE-TPGS/EL and LE-TPGS/SolP vs. LE-HS/SolP) and for the binary mixture based on EL (LE-HS/EL vs. LE-EL/SolP); exclusively, the TPGS/HS mixture allowed a high LE-In, of up to 0.253 mg/mL.

Probably, the molar fraction of TPGS in the bulk (α_1_), associated with the maintenance of an appropriate grade of hydrophilicity gained due to the presence of HS (HLB = 14–16) rather than SolP (HLB = 16), allowed a synergistic effect that was able to promote LE solubility.

These results, together with other macroscopic properties including both the optimal dimensional characteristics of the nanomicelles (13.57 nm) and their physical characteristics in terms of transparency and filterability (see Table 2), which represent two crucial points for an ophthalmic solution, led to the selection of the TPGS/HS binary combination.

### 2.3. Optimisation of LE-TPGS/HS Nanomicelles

In the attempt to optimise the amount of the TPGS/HS mixture, a study on the influence of its concentration on LE solubility was performed. 

**Figure 2:**
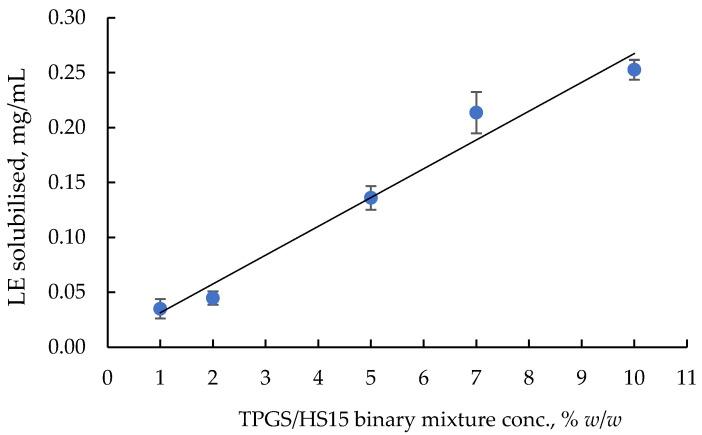
Amount of LE solubilised (LE-In) in PBS in presence of different concentrations of the 1:1 ratio of TPGS/HS of the binary mixture (mean ± SD, n = 3).

The results are summarised in Figure 2 where LE-In for different concentrations of TPGS/HS binary mixture in a (1:1) weighted ratio is reported. A quite linear correlation is evidenced by the experimental points with a tendency to reach a plateau for the highest concentration of the polymeric surfactant. However, statistically significant differences in LE-In between 7.0% and 10% *w*/*w* concentrations of the (1:1) TPGS/HS binary mixture (*p* < 0.05, *t*-test unpaired analysis) were observed. Probably, this positive effect on the solubility, rather than being due to the type of selected surfactant, could have been due to an interaction between the chains of these molecules with a probable synergism.

The presence of a synergism with positive interactions between the TPGS and HS polymeric surfactants can be demonstrated by applying Clint’s model that allows a study of the ideal nature of mixed micelles. Any deviation of CMC_exp_ from the ideal CMC of the mixed micelles (${\mathrm{CMC}}_{\mathrm{mix}}^{*}$) involved the mutual interactions among surfactant components. A negative deviation (CMC_mix_ < ${\mathrm{CMC}}_{\mathrm{mix}}^{*}$) represents synergistic interactions whereas a positive deviation (CMC_mix_ > ${\mathrm{CMC}}_{\mathrm{mix}}^{*}$) indicates antagonistic ones. In addition, a more in-depth interpretation of the possible interaction in the LE-TPGS/HS nanomicelles arises from the calculation of the β parameter according to Equations (4) and (5) reported in Section 3.6.4. The CMC_TPGS/HS_ value obtained by applying the pendant drop method was 0.0983 mM and the interaction parameter between the polymeric surfactant-building units (β_TPGS/HS_) was equal to −0.1322 (see Table 4). This negative value confirmed the ability of the polymeric surfactants to interact, favouring LE dissolution in the lipophilic core of the nanomicelles.

### 2.4. Physico-Chemical and Biological Characterisation of LE-Loaded Mixed Nanomicelles (LE-TPGS/HS)

**Figure 3:**
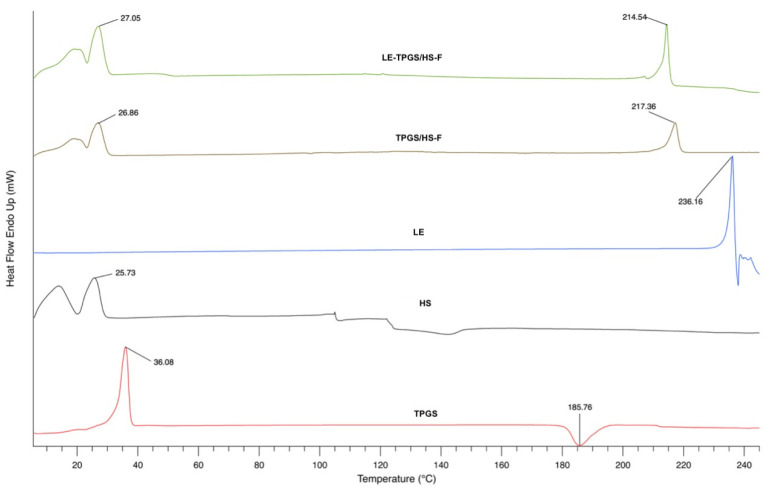
DSC thermograms of TPGS (red line) and HS (black line) surfactant polymers, LE pure drug (blue line), TPGS/HS-F mixed freeze-dried nanomicelles (brown line) and LE-TPGS-F drug-encapsulated freeze-dried nanomicelles (green line).

The results from the physico-chemical characterisation of the LE-TPGS/HS nanomicelles via DSC are in Figure 3, where the thermograms of the freeze-dried selected nanomicelles (LE-TPGS/HS-F and TPGS/HS-F) are reported with those of each polymeric surfactant and of the pure drug. 

Endothermic melting peaks were detected at 236.16 °C, 15.2 °C and 25.73 °C, and 36.08 °C, for LE, HS and TPGS, respectively, which matched their typical melting points (LE, m.p. = 220–224 °C from PubChem data; Kolliphor^®^ HS-15 and Kolliphor^®^ TPGS, m.p. = 25–30 °C and 36–41 °C, respectively, from BASF).

The thermogram of both nanomicelles exhibits a series of endothermic peaks at low temperatures (26.86 °C and 27.05 °C for TPGS/HS-F and LE-TPGS/HS-F, respectively), which do not correspond to those of the original substances and the presence of an endothermic peak at 217.36 °C and 214.54 °C for TPGS/HS-F and LE-TPGS/HS-F, respectively. In particular, the disappearance of the endothermic LE transition in LE-TPGS/HS-F is attributed to its encapsulation into the lipophilic core of the nanomicelles and to the interactions with the polymeric surfactants [35]. Even the disappearance of the endothermic peak at 36.08 °C related to TPGS could be a further confirmation of the rearrangement of the polymers into nanostructures able to maintain a high amount of the drug in solution. 

**Figure 4:**
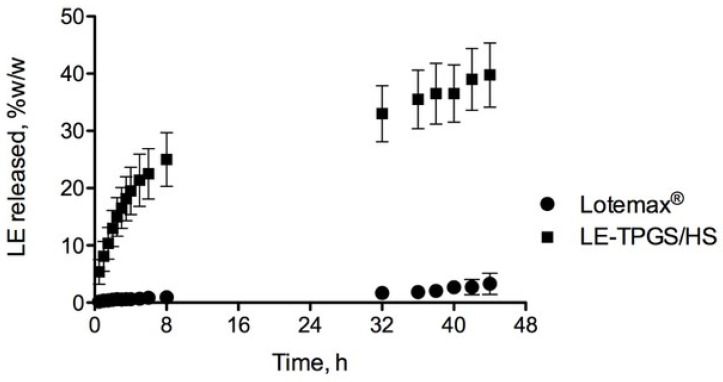
In vitro drug release profiles of LE-TPGS/HS and commercial-suspension Lotemax^TM^ eye drop (mean ± SD, n = 3).

The results of the LE in vitro release from the MixNano formulations performed under sink conditions via the dynamic dialysis method are shown in Figure 4. 

More than 20% of LE was released from LE-TPGS/HS within the first 5 h in the PBS buffer, while a very low percentage of the drug was found in the case of the commercial-suspension Lotemax™ eye drops (0.5% LE). Despite the scarce aqueous solubility of LE, the nanomicellar carrier favoured its solubilisation, sustaining its release in the dissolution medium for several hours with about 40% of LE being detected after 48 h. The dissolution profile seems to be a typical diffusive profile similar to those obtained for other nanomicellar systems encapsulating insoluble drugs [36].

**Figure 5:**
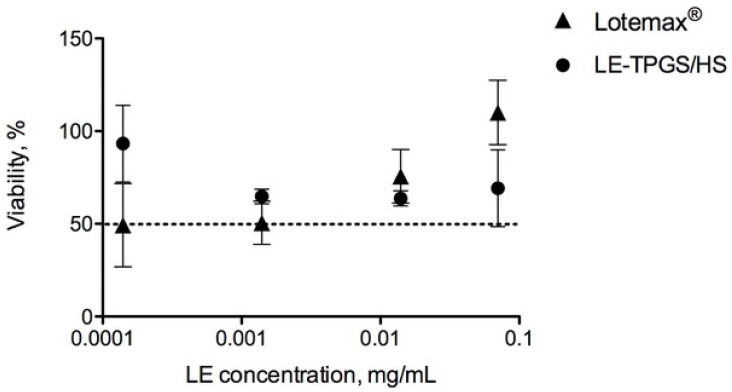
Cytotoxicity of RCE of LE-TPGS/HS and commercial-suspension Lotemax™ eye drop (mean ± SD, n = 3).

The results of the cytotoxicity study, which was aimed at highlighting any possible toxicity of the polymeric surfactants, are displayed in Figure 5, where the percentage of cell viability, measured for both LE-TPGS/HS and the Lotemax™ eye drop, is reported as a function of the concentration of LE, that represents the common ingredient. The treatment of RCE with Lotemax™ showed slight cytotoxicity, albeit with a high experimental viability, by reducing the LE concentration. In any case, cell viability remained above 50% for all the tested concentrations.

The slight increase in cytotoxicity of the nanomicellar formulation at the highest concentrations was probably linked to the presence of polymeric surfactants. In fact, the decrease in the polymeric surfactant concentration observed in the more diluted samples increased cell viability, which ultimately was about 100% (0.0001% LE concentration). 

A crucial need concerns the evaluation of the stability of the nanomicellar formulation. The good stability of the selected LE-TPGS/HS nanomicellar formulation was demonstrated in terms of size, LE recovery and clarity, as summarised in Table 5.

In fact, the appearance of the LE-TPGS/HS nanomicelles remained remarkably transparent, with a constant size up to 30 days and with a slight increase in mean diameter after 90 days (from 12.97 to 13.87 nm).

## 3. Materials and Methods

### 3.1. Chemicals

The following materials were used as received: loteprednol etabonate (LE), kindly given by Farmabios (Pavia, Italy); Macrogol 15 hydroxy stearate (HS, Kolliphor^®^ HS-15); polyvinyl caprolactam–polyvinyl acetate–polyethylene glycol copolymer (SolP, Soluplus^®^); Polyoxyl-35 hydrogenate castor oil (EL, Kolliphor^®^-EL), kindly provided by BASF (Ludwigshafen, Germany); d-α-Tocopherol polyethylene glycol succinate (TPGS, Isodel^®^ Vitamin E TPGS 1000), provided by PMC Isochem (Vert-Le-Petit, France); Lotemax™, provided by Bausch & Lomb IOM S.p.A (Vimodrone, Italy). All other chemicals and solvents were of analytical grade. Water was purified via reverse osmosis using the MilliQ apparatus (Millipore^®^, Milan, Italy). 

### 3.2. Cell Cultures

The abbit corneal epithelial cell line (RCE, European Cell Culture Collection N° 95081046, ECACC, Salisbury, UK) was used for the cytotoxicity test. The growth medium had the following composition: Dulbecco’s modified Eagle’s medium (DMEM) with Ham’s nutrient mixture F12 (1:1) with addition of L-glutamine (1% *v*/*v*), penicillin (100 IU/mL), streptomycin (0.1 mg/mL), amphotericin B (0.25 μg/mL), foetal bovine serum (15% *v*/*v*) (Gibco Invitrogen S.r.l., Milan, Italy), epidermal growth factor (10 ng/mL), and insulin (5 mg/mL) (Sigma Chemical Co., St. Louis, MO, USA).

### 3.3. HPLC Analytical Method

The detection of the amount of LE was carried out via HPLC. The system consisted of a LC-6AS pump, a SPS-10AV detector, a C-R4A integrating system, and a 20 µL Rheodyne injector sample loop (Shimadzu Italia s.r.l., Milan, Italy). The mobile phase, delivered at a flow rate of 1.0 mL/min (pressure about 78–80 kgf) in a 5 µm reversed-phase C18 column (Kinetex Phenomenex EVO C18 100A, 100 × 4.6 mm, Phenomenex, Torrance, CA, USA) was a mixture of methanol and water at a 65:35 ratio. The detection wavelength was 254 nm and the retention time of LE under these conditions was 6.8 min. 

The amount of LE in the samples was determined via comparison with an external standard curve obtained via the dilution of a standard solution of LE in methanol with PBS, applying the least squares linear regression analysis (Prism 8 software, GraphPad Software Inc., San Diego, CA, USA).

The external standard curve was performed in the 0.1–10.0 μg/mL LE concentration range and the R-squared (R^2^) value was 0.9982.

### 3.4. Solubility Study of Loteprednol Etabonate

A LE solubility study was performed by adding an excess of the drug (about 10 mg) and an exactly weighted amount of the different solubilising polymers (TPGS, HS, SolP, and EL) to 10.0 g of PBS, at pH 7.4. The concentration of each polymer was 5.0% *w*/*w* in the final suspensions. The suspensions were stoppered in a glass vial and maintained under magnetic stirring for 12 h at room temperature to allow the complete dispersion of the solubilising polymeric surfactants and to favour LE dissolution. Then, the samples were filtered through a 0.2 μm membrane (Phenex^®^ RC Syringe filter, Phenomenex, Torrance, CA, USA) to remove the unloaded drug, aggregates, and other foreign particulates. The amount of LE (LE-In) solubilised by the different solubilising agents was determined via HPLC analysis. For the analysis, aliquots (100 μL) of each preparation were diluted with methanol to ensure the complete solubilisation of the incapsulated/loaded LE. The physico-chemical characteristics of the selected solubilising polymeric surfactants (HLB and CMC) obtained from the literature data are reported in Table 6. The polymers were chosen by taking into account both their capability to solubilise LE, as observed in preliminary experiments (data no reported), and their well-known enhancer effect on drug permeation into tissues, which are useful for different types of applications, including ocular use [37,38,39,40,41,42].

### 3.5. Preparation of LE-Loaded Mixed Nanomicelles (LE-MixNano)

LE-MixNano was prepared by using 10% *w*/*w* of the (1:1) binary mixture of the selected solubilising polymeric surfactants, following the previously reported procedure. In detail, appropriate amounts of surfactants and LE (0.01 g) were weighted and PBS was added as a solvent to the blend in an amount of up to 10.0 g. The final LE dispersions (0.1% *w*/*w*), after being stirred for 12 h at room temperature, were filtered through 0.2 μm filters to remove the unloaded drug, aggregates, and other foreign particulates. 

The same procedure was used to prepare empty MixNano exclusively based on a couple of polymeric surfactants (TPGS/HS).

### 3.6. Characterisation of LE-Loaded Mixed Nanomicelles (LE-MixNano)

#### 3.6.1. Size Distribution and Polydispersity Index analysis 

The average hydrodynamic diameter (D_h_) and polydispersity index (PI) of LE-MixNano were determined using dynamic light scattering (DLS) analysis (Beckman Coulter^®^ N4 Plus, Beckman Coulter s.r.l, Milan, Italy). Five minutes before the DLS measurements, each sample was adequately diluted with ultrapure water, previously filtered through a 0.45 μm pore size filter (Phenex^®^ RC Syringe filter, Phenomenex, Torrance, CA, USA), choosing the final concentration to reach a measurement intensity ranging from 5 × 10^4^ to 1 × 10^6^ counts per second (cps). D_h_ and PI for each LE-MixNano preparation were measured at 20 °C with 6 runs of three different samples at an angle of 90° [35] and a run time of 200 s.

#### 3.6.2. Determination of the Amount of Solubilised LE (LE-In) and of LE Encapsulation Efficiency (LE-EE) in MixNano Formulations

LE-In, by using the different surfactant binary mixtures, was determined via HPLC after diluting aliquots (100 μL) of each LE-MixNano preparation with methanol (20.0 mL). Each analysis was repeated on three different samples.

The determination of LE-In allowed the calculation of LE encapsulation efficiency (LE-EE) applying the following equation:

LE-EE (% *w*/*w*) = (weight of LE in LE-MixNano × 100)/(weight of feed LE) (1)

#### 3.6.3. Evaluation of the Clarity and Filterability of LE-MixNano Formulations

To select of the most suitable ophthalmic formulations, a score regarding the degree of clarity of the formulations was assigned according to the partially modified scale of Makwana et al. [43] reported in Table 7. The scores were obtained via visual inspection under bright light against a black and white background, with the swirling of the LE-MixNano formulations.

In addition, the facility of filtration of LE-MixNano formulations was also tested by using a 0.2 μm filter. The filtration process represents the more usual method to sterilise ophthalmic formulations and suitable filterability can be an added benefit in developing a preparation process. A score based on the degree of filterability was assigned, as described in Table 8. 

#### 3.6.4. Investigation on the Synergism between the Different Surfactant Mixtures

To determine the presence of synergistic interactions between the different polymeric surfactants of the binary mixtures and the possible deviation from the ideal mixing behaviour of polymeric surfactants in micelles, the theoretical values of ${\mathrm{CMC}}_{\mathrm{mix}}^{*}$ and the interaction parameter (β_1,2_) between a couple of polymeric surfactants were calculated. 

The calculation of the theoretical ${\mathrm{CMC}}_{\mathrm{mix}}^{*}$ values for the different binary combinations was carried out by applying Clint’s theory, knowing the individual CMC for each single surfactant of the binary mixtures and the mole fractions of surfactants. The ${\mathrm{CMC}}_{\mathrm{mix}}^{*}$ values were obtained using Equation (2):

(2) $$\frac{1}{{\mathrm{CMC}}_{\mathrm{mix}}^{*}}=\frac{\alpha_{1}}{{\mathrm{CMC}}_{1}}+\frac{\left(1-\alpha_{1}\right)}{{\mathrm{CMC}}_{2}}$$

where, CMC_1_ and CMC_2_ are the critical micellar concentration of the first and second surfactant, respectively, and α_1_ is the mole fraction of the first polymeric surfactant of the binary mixture.

Furthermore, the divergence from the ideal behaviour of the micellar systems was defined by calculating the mole fraction of the first surfactant ($X_{1}^{*}$) in the mixed micelles in the ideal state, applying Equation (3):

(3) $$X_{1}^{*}=\frac{\alpha_{1}{CMC}_{2}}{\alpha_{1}{\mathrm{CMC}}_{2}+\left(1-\alpha_{1}\right){\mathrm{CMC}}_{1}}$$

In addition, a very useful formula to calculate the molecular interactions and the extent of synergistic interaction in a non-ideal model is provided by the iterative Rubingh’s procedure [44,45]. If the behaviour of mixed micelles deviates considerably from the ideal mixing behaviour, Rubingh’s model can be applied, obtaining a X_1_ value that represents the mole fraction of the first polymeric surfactant in the mixed micelles (Equation (4)):

(4) $$\frac{{\left(X_{1}\right)}^{2}\ln\left(\alpha_{1}{\mathrm{CMC}}_{\mathrm{mix}}/X_{1}{\mathrm{CMC}}_{1}\right)}{{\left(1-X_{1}\right)}^{2}\ln\left[\left(1-\alpha_{1}\right){\mathrm{CMC}}_{\mathrm{mix}}/\left(1-X_{1}\right){\mathrm{CMC}}_{2}\right]}=1$$

The extent of deviation from the ideal mixing behaviour is confirmed by a dimensionless parameter, β, which can be calculated using Equation (5):

(5) $$\beta_{1,2}=\frac{\ln\left(\frac{\alpha_{1}{\mathrm{CMC}}_{mix}}{X_{1}{CMC}_{1}}\right)}{{\left(1-X_{1}\right)}^{2}}$$

where X_1_ represents the mole fraction of component 1 in the total mixed micellar system, α_1_ is the mole fraction of the first surfactant in the total bulk of the binary system, CMC_mix_ is the CMC of the system, while CMC_1_ and CMC_2_ are the CMCs of each single surfactant.

#### 3.6.5. Influence of Concentration of TPGS/HS Mixture on LE Solubilisation

To optimise the percentage of the selected binary polymeric mixture (TPGS/HS, 1:1 weight ratio) to be used, concentrations lower than 10% *w*/*w* of the selected binary mixture (1.0, 2.0, 5.0, 7.0% *w*/*w*) were evaluated to solubilise at least the same amount of the drug. The preparation was performed following the already reported procedure (Section 3.4), by adding exactly weighted amounts of the TPGS/HS (1:1 weight ratio) binary mixture and LE (0.1% *w*/*w*) to 10.0 g of PBS. The analysis via HPLC was carried out for three samples of each binary mixture percentage.

#### 3.6.6. Determination of Experimental Critical Micellar Concentration (CMC_exp_)

The optical method of pendant drop was chosen to measure the surface tension values of both single polymeric surfactant dispersions and the selected TPGS/HS (1:1 weight ratio) binary mixture. The surface tension measurements were performed at room temperature using the OCA15 optical contact angle measuring system (DataPhysics Instrument, Filderstadt, Germany) [46]. The system consisted of a high-resolution CCD video camera and a six-fold-power-zoom lens with integrated fine focusing that allowed us to capture the profile of the sessile droplets obtained for each polymeric dispersion. The images were recorded and analysed using SCA20 software (DataPhysics).

Briefly, the droplets, having an exactly measured the volume, ranging between 20–30 μL, were produced by using a capillary needle, placed on top of the testing cell and released at 50 μL/min. Once the droplet was formed, its detection level was controlled by adjusting the brightness and the contrast level by using a mounted camera able to analyse, record, and capture the image of each drop of the different surfactant samples. The surface tension value was determined using the Laplace–Young equation based on the complete shape of the pendant droplet as suggested by Berry et al. [47]. The tested concentrations ranged from 1.0 × 10^−4^ to 1.0% *w*/*w* and from 2.0 × 10^−3^ to 0.1% *w*/*w*, for each single polymeric surfactant dispersion and for the TPGS/HS (1:1) binary mixture, respectively. Ten measurements were performed for each preparation.

### 3.7. Freeze-Drying of the Selected LE-MixNano Formulation

TPGS/HS and LE-TPGS/HS were subjected to a freeze-drying process obtaining dry products useful for DSC characterisation. Different aliquots of the formulations (150–200 μL) were collected in different vials and underwent a controlled freeze-drying cycle. The cycle consisted first of a freezing process (pressure, 400 torr; freezing temperature, −38 °C; freezing rate, 0.6 °C/h; extra freeze time, 120 min), followed by primary drying (pressure, 100 torr; temperature, −38 to 0 °C; rate, 2.1 °C/h) and secondary drying (pressure, 50 torr; temperature, up to 25 °C; rate, 5.0 °C/h; extra drying, 27 °C for 60 min) [35]. The freeze-dried nanomicellar formulations (TPGS/HS-F and LE-TPGS/HS-F) were stored at room temperature in desiccators until use.

### 3.8. Thermal Analysis of the Freeze-Dried Formulations by Differential Scanning Calorimetry (DSC)

DSC analysis, of both the freeze-dried MixNano formulations (TPGS/HS-F and LE-TPGS/HS-F) and the raw materials (LE, HS and TPGS), was performed by using a differential scanning calorimeter (DSC 6, PerkinElmer, Milan, Italy). The samples (1.5–2.0 mg) were placed and sealed in a flat-bottomed aluminium pan and heated at a constant rate of 5 °C/min under nitrogen purge gas at a rate of 20 mL/min. The range of temperature tested was from 5 °C to 250 °C. The thermal profiles were recorded with Pyris Instrument Managing Software (Version 3.8, Perkin Elmer, Milan, Italy), and analysis was performed using IgorPro^®^ software (Version 7.0, WaveMetrics Inc., Portland, OR, USA). 

### 3.9. Cytotoxicity Assay

A cytotoxicity test was performed on a rabbit corneal epithelial cell line (RCE) using the ready-to-use cell proliferation reagent WST-1 (cat no. 1644807, Roche Diagnostics GmbH, Germany). The assay is based on cleavage of the tetrazolium salt WST-1 by mitochondrial enzymes to produce formazan salt, which is completely soluble in water with cherry-red colouration. Only viable cells can reduce WST-1, whose staining is therefore proportional to the viable cell number. The RCE cells were plated at 3 × 10^5^ cells/well, in 96-well microtiter plates and treated with LE-TPGS/HS and a commercial Lotemax™ eye drop, which was chosen as a reference. 

The cytotoxicity protocol has been previously developed by the authors and extensively published [35,42,48,49]. Both formulations were suitably diluted in a growth medium to obtain a LE concentration in the range between 0.07 and 1.4 × 10^−2^ mg/mL. After 15 min of exposure, the formulations were removed, and the cells were washed twice with DMEM/F12. Then, 100 μL of a fresh growth medium and 10 μL of the reagent WST-1 were added to each well. The cells were incubated for 2 h at 37 °C, the microplates were thoroughly shaken for 30 s, and the absorbance was measured at 450 nm using the microplate reader (Asys UVM 340, Biochrom Ltd., Cambridge, UK). The use of a 2 hr incubation period was based on a series of preliminary experiments. The background absorbance was measured from the wells containing only the dye solution and the culture medium.

The results were expressed as the percentage absorbance of the treated vs. untreated control wells according to Equation (6):

(6) $$\mathrm{Cell}\;\mathrm{viability}\;\%=\frac{\mathrm{Treated}\;\mathrm{Abs}}{\mathrm{Control}\;\mathrm{Abs}}\times 100$$

### 3.10. Physico-Chemical Stability of the Selected Nanomicellar Formulation

To evaluate the physico-chemical stability of LE, three different batches of LE-TPGS/HS were prepared following the method described in Section 3.5. After preparation, two aliquots of each batch were packaged into different crimp vials and incubated at 4 °C for 90 days. The concentration of LE in the samples was analysed over time via the HPLC method after appropriate dilution with methanol. The LE detected in the three different samples for each time point was reported as a percentage of the initial amount.

The evaluation of the physical stability of the formulation was carried out by determining the size distribution, polydispersity index and clarity over the time according to the procedures reported in Section 3.6.1 and Section 3.6.3.

### 3.11. In Vitro LE Release Studies 

The in vitro LE release profile from LE-TPGS/HS was investigated via the dynamic dialysis method using the Lotemax™ eye drop as reference. 

Five hundred microliters of the formulations were transferred into a closed dialysis bag (MWCO 3500 Da, Spectra/Pore 3 Dialysis Membranes, Spectrum labs, Breda, NL, USA) and placed in borosilicate vials filled with 5.0 mL of PBS. The receiving medium was maintained at 32 °C to simulate the temperature of the ocular surface and stirred with a paddle running at 20 rpm. An amount of 1.0 mL of the receiving phase was withdrawn for HPLC analysis every 30 min and replaced with the fresh buffer to maintain the sink conditions. All experiments lasted 5 h and were performed in triplicate.

## 4. Conclusions

This work provides scientific evidence of the efficacy of a novel TPGS/HS binary mixture that is able to improve LE solubility by self-assembling in drug-loaded mixed nanomicelles. LE represents an advantageous topical corticosteroid with a reduced risk of adverse reactions, being transformed into inactive metabolites after exerting its therapeutic effects in the treatment of inflammatory diseases of the external (dry eye disease) and/or anterior segment of the eye, as well as postoperative inflammation following ocular surgery. 

The novel LE-TPGS/HS nanomicelles, containing 0.253 mg/mL of the drug, have small dimensions, a uniform size distribution, appreciable stability, and capability to promote sustained LE release. 

Although a complete pharmacodynamic study has not yet been performed, the cytotoxicity data on RCE cells confirm the possible ocular application of this nanomicellar system. The lack of a significant cytotoxic effect on a sensitive cell line warrants further studies aimed at demonstrating the therapeutic efficacy of the novel formulation.

The main commercial products containing LE are suspensions such as Lotemax^®^ and Alrex^®^, although more advanced technologies have been exploited to improve the drug efficacy for the back of the eye, as in case of Eysuvis™, which is based on custom-engineered mucus-penetrating-particle (MPP) technology, or of the submicron nanosuspension added to mucoadhesive gel [50]. To date, the biopharmaceutical properties of topical ocular suspensions are still poorly understood. In general, only the fraction of the suspended particles that dissolve in tears are responsible for a therapeutic effect; in fact, it is known that the presence of smaller particles increases the ocular absorption of the drug from equiviscous suspensions to up to 1.6–2.3 fold [51]. Nevertheless, the bioavailability of an ocular suspension is strictly correlated to the presence of specific excipients able to modify the residence time of the suspended drugs, as well as the size of the drug particles. In view of the fact that the drug is absorbed once it is solubilised, the administration of a solution avoids these critical issues; in fact, the bioavailability of a solution is generally greater than that of a suspension, obtaining the same therapeutic effect with a lower dose administered [52]. Therefore, an advantageous pharmacokinetic profile could be obtained with the selected TPGS/HS mixed nanomicellar formulation with a 0.253 mg/mL LE concentration. The combination of the two polymeric surfactants (TPGS than HS) could promote drug corneal permeation with the enhancement of ocular bioavailability [38,41]. 

## Figures and Tables

**Table 1:** Amount of solubilised LE (LE-In), encapsulation efficiency (LE-EE), size distribution (D_h_), polydispersity index (PI), and clarity and filterability of the developed formulations (mean ± SD, n = 3).

Types of LE-MixNano	LE-In (mg/mL)	LE-EE (% *w*/*w*)	D_h_ (nm)	PI	Clarity	Filterability
LE-TPGS/HS	0.253 (±0.007)	25.3 (±0.7)	13.57 (±0.49)	0.271 (±0.110)	+++	++
LE-TPGS/SolP	0.192 (±0.003)	19.2 (±0.3)	17.93 (±0.91)	0.343 (±0.150)	+	+
LE-TPGS/EL	0.200 (±0.010)	20.1 (±1.0)	12.03 (±0.25)	0.303 (±0.091)	++	++
LE-HS/EL	0.129 (±0.002)	12.9 (±0.2)	13.47 (±0.31)	0.145 (±0.115)	++	++
LE-HS/SolP	0.176 (±0.007)	17.6 (±0.7)	85.13 (±0.81)	0.507 (±0.020)	+	+
LE-EL/SolP	0.121 (±0.004)	12.1 (±0.4)	68.27 (±1.63)	0.471 (±0.034)	+	+

**Table 2:** Calculated CMC experimental values (CMC_exp_) (mean, n = 10) and theoretical CMC (CMC_theor_) for each single polymeric surfactant.

Polymeric Surfactant	CMC_exp_ (mM)	CMC_exp_ (% *w*/*w*)	CMC_theor_ (mM)	CMC_theor_ (% *w*/*w*)
TPGS	15.33 × 10^−2^	2.32 × 10^−2^	13.22 × 10^−2^	2.00 × 10^−2^
SolP	7.12 × 10^−5^	0.84 × 10^−3^	6.44 × 10^−5^	7.60 × 10^−4^
EL	163.06 × 10^−2^	2.22 × 10^−2^	146.90 × 10^−2^	2.00 × 10^−2^
HS	7.83 × 10^−2^	1.22 × 10^−2^	8.74 × 10^−2 #^	1.25 × 10^−2 #^

^#^ Mean value calculated from the two extreme values of the range reported in the BASF technical data sheet (CMC = 0.005–0.02%).

**Table 3:** Ideal CMC (${\mathrm{CMC}}_{\mathrm{mix}}^{*}$) and molar fraction ($X_{1}^{*}$) values calculated for the different binary mixtures.

Binary Mixtures	α_1_	${\mathrm{CMC}}_{\mathrm{mix}}^{*}$ (mM)	$X_{1}^{*}$
TPGS/HS	0.4859	0.1027	32.56 × 10^−2^
TPGS/SolP	0.9873	0.0054	2.31 × 10^−4^
TPGS/EL	0.0825	0.9082	44.78 × 10^−2^
HS/EL	0.0869	0.5988	62.47 × 10^−2^
HS/SolP	0.9880	0.0055	4.51 × 10^−4^
EL/SolP	0.9989	0.0595	2.18 × 10^−5^

**Table 4:** Experimental and theoretical parameters for the LE-TPGS/HS nanomicelles: ${\mathrm{CMC}}_{\mathrm{mix}}^{*}$, ideal CMC; CMC_mix_, experimental CMC; $X_{1}^{*}$, theoretical molar fraction of TPGS in LE-TPGS/HS nanomicelles; X_1_, calculated molar fraction of TPGS in LE-TPGS/HS nanomicelles; β_TPGS/HS_, interaction parameter between the polymeric surfactant-building units.

Type of Nanomicelles	CMC_TPGS_ (mM)	CMC_HS_ (mM)	CMC*_TPGS/HS_ (mM)	CMC_TPGS/HS_ (mM)	X_1TPGS_	$X_{1}^{*}$ _TPGS_	β_TPGS/HS_
LE-TPGS/HS	0.1533	0.0783	0.1027	0.0983	0.3398	0.3256	−0.1322

**Table 5:** Storage stability of the selected LE-TPGS/HS nanomicellar formulation at 4 °C; size distribution (D_h_) and polydispersity index (PI) (mean ± SD, n = 3).

Time Days	D_h_ nm	PI	LE Recovered % *w*/*w*	Clarity
0	12.93 (±0.61)	0.199 (±0.020)	100.00 (±0.95)	+++
30	12.97 (±0.42)	0.220 (±0.081)	96.32 (±3.77)	+++
90	13.87 (±3.39)	0.869 (±0.592)	94.82 (±0.77)	+++

**Table 6:** Physico-chemical characteristics of the amphiphilic polymers: hydrophilic lipophilic balance (HLB) and critical micellar concentration (CMC).

Type of Polymer	HLB	CMC_theor_ % *w*/*w*
TPGS	13	0.02 ^&^
HS	14–16	0.005–0.02 ^§^
SolP	14	0.00076 ^§^
EL	12–14	0.02 ^§^

^&^ obtained from PMC Isochem. ^§^ obtained from BASF data sheet.

**Table 7:** Score of the grade of clarity of LE-MixNano formulations.

Clarity	Grade
Cloudy: the formulation is a milky-white solution.	+
Opaque: the formulation is colourless and slightly opalescent with suspended particles.	++
Transparent: the formulation is completely clear.	+++

**Table 8:** Score of the grade of filterability of LE-MixNano formulations.

Filterability	Grade
Hard to filter: the formulation presents strong resistance to filtration, clogging the filter.	+
Filterable: the formulation is easily filtrable without any impediment.	++

## Data Availability

Data is contained within the article.
